# FROM TWINS TO DIGITAL TWINS: LLM-DRIVEN COGNITIVE PERFORMANCE PREDICTION

**DOI:** 10.1093/geroni/igae098.4197

**Published:** 2024-12-31

**Authors:** Anqing Zheng, Daniel Gustavson, Robin Corley, Chandra Reynolds

**Affiliations:** University of Colorado Boulder, Boulder, Colorado, United States; University of Colorado Boulder, Boulder, Colorado, United States; University of Colorado Boulder, Boulder, Colorado, United States; University of Colorado Boulder, Boulder, Colorado, United States

## Abstract

Human digital twins, virtual representations of individuals that simulate one or more aspects of human behavior and characteristics, have gained increasing attention for their potential applications in training optimization and personalized interventions. This study explores the use of Large Language Models (LLMs) with in-context learning to simulate cognitive performance, leveraging the power of real human sibling pairs. Our study aims to assess the accuracy of these simulated representations by comparing their predictive power against actual performance and their siblings’ performance across various cognitive domains in those approaching midlife. We utilize the CATSLife dataset of 1,040 individuals from 505 sibships (including twins and non-twin pairs, ages 28-49). Our dataset encompasses over 60 characteristics with empirical associations with cognitive maintenance, spanning across lifestyle, psychosocial, environmental, and demographic domains, serving as the foundation for simulating cognitive performance. For evaluation, we employ intraclass correlation coefficients to quantify similarities between sibling-sibling, individual-simulation, and sibling-simulation pairs. Preliminary analyses reveal strong cognitive performance correlations among monozygotic pairs (MZ; rs =.37~.85), establishing a benchmark for our simulations. We expect our LLM-based simulation will outperform traditional statistical approaches in predicting individual cognitive performance, while remaining within the bounds of observed MZ similarities. By comparing sibling-sibling and sibling-simulation similarities, we aim to demonstrate a novel evaluation metric for human digital twins and provides insights into the capabilities of advanced computational methods in predicting individual cognitive performance. This approach could open avenues for developing more precise, personalized strategies in cognitive health management and targeted cognitive enhancement interventions.

